# Facilitators and barriers to atrial fibrillation screening in primary care: a qualitative descriptive study of GPs in primary care in the Republic of Ireland

**DOI:** 10.3399/BJGPO.2022.0110

**Published:** 2023-05-03

**Authors:** Aileen Callanan, Farshid Bayat, Diarmuid Quinlan, Patricia M Kearney, Claire M Buckley, Susan M Smith, Colin P Bradley

**Affiliations:** 1 School of Public Health, University College Cork, Cork, Republic of Ireland; 2 Department of General Practice, University College Cork, Cork, Republic of Ireland; 3 School of Medicine and Health, University College Cork, Cork, Republic of Ireland; 4 Irish College of General Practitioners, Dublin, Republic of Ireland; 5 Department of Public Health and Primary Care, Trinity College, Dublin, Republic of Ireland

**Keywords:** screening, primary health care, atrial fibrillation, qualitative research

## Abstract

**Background:**

Atrial fibrillation (AF), the most common cardiac arrhythmia, is a major risk factor for stroke. AF is often asymptomatic, making it difficult to diagnose. Globally, stroke is a leading cause of morbidity and mortality. Opportunistic AF screening has been recommended in clinical practice within the Republic of Ireland (RoI) and internationally, though the optimal mode and location remains under investigation. Currently, there is no formal AF screening programme. Primary care has been proposed as a suitable setting.

**Aim:**

To identify the facilitators and barriers to AF screening in primary care from the perspective of GPs.

**Design & setting:**

A qualitative descriptive study design was adopted. Fifty-four GPs were invited from 25 practices in the RoI to participate in individual interviews at their practices. Participants were from both rural and urban locations.

**Method:**

A topic guide was developed to guide the interview content towards identification of facilitators and barriers to AF screening. The interviews were conducted in person, audio-recorded, transcribed verbatim, and analysed using framework analysis.

**Results:**

Eight GPs from five practices participated in an interview. Three GPs, two male and one female, were recruited from two rural practices and five GPs, two male and three female, were recruited from three urban practices. All eight GPs expressed a willingness to engage in AF screening. Time pressures and the need for additional staff to support were identified as barriers. Programme structure and patient awareness campaigns and education were identified as facilitators.

**Conclusion:**

The findings will help to anticipate barriers to AF screening and aid the development of clinical pathways for people with or at risk of AF. The results have been integrated into a pilot primary care-based screening programme for AF.

## How this fits in

This is the first study, to the authors' knowledge, to qualitatively investigate facilitators and barriers to AF screening from the perspectives of GPs in primary care in the RoI. The results can be used to inform stakeholders regarding national AF screening programmes.

## Introduction

AF is the most common cardiac arrhythmia worldwide.^1^ Its incidence increases significantly with age, and it is estimated to affect approximately 11% of the Irish population aged ≥65 years.^2^ A 2022 report of chronic disease burden in the RoI estimated the prevalence of AF at 3.1% for people aged 60–69 years, 5.0% for people aged 70–74 years, and 7.4% for people aged ≥75 years.^3^ The first report of the structured chronic disease management programme reported 262 109 registered diagnoses, with 16.5% of these for AF.^4^ AF is frequently asymptomatic, making it difficult to diagnose.^5^ The irregular beating of the heart in AF enables blood clots to form, increasing the risk of stroke five-fold.^1^ AF is estimated to be responsible for approximately 20–30% of strokes, and AF strokes are frequently severe or fatal.^2^ Importantly, early detection of AF, with initiation of oral anti-coagulation treatment, reduces stroke risk by up to two-thirds.^5^

Screening for AF has been recommended both nationally and internationally although, to date, there are no formal national AF screening programmes. The European Society of Cardiology (ESC) guidelines recommend opportunistic screening for patients aged ≥65 years and systematic screening for patients aged ≥75 years via pulse palpation or electrocardiogram (ECG) rhythm strip.^6^ The current recommendation to confirm a diagnosis of AF is a 12-lead ECG or single-lead ECG recording of ≥30 seconds.^7^ There are a range of other approaches that could be utilised in AF detection and could be included in a screening approach, including pulse palpation, mobile ECG devices, 12-lead ECG devices, and personal health monitoring devices. Mobile ECG devices have been proposed as the preferred AF screening tool^8^ and have been found to be more accurate than pulse palpation, with higher specificity.^9^ Studies have estimated the sensitivity of one-lead ECG to be 94%, with specificity at 97%, and they have been found to be superior to pulse palpation.^10^ While there is international consensus that AF diagnosis is beneficial, the optimal detection mode depends on individual test performance and specific country and health system characteristics.^8^ Owing to the paroxysmal nature of AF, single-time point screening can miss cases where the patient is in sinus rhythm at the time of assessment.^7^ However, this is often the nature of a screening programme, where healthy populations are being screened for undiagnosed disease and, in the case of AF screening, repeat screening would be conducted at regular intervals.

Previous studies conducted in the RoI and across Europe have found AF screening across various modes, including pulse palpation, mobile ECG, and wearable technology to be cost-effective.^11^ A health technology assessment of AF screening in the RoI estimated a decrease in stroke incidence of 1.9% after 5 years. The estimated incremental cost-effectiveness ratio (ICER) was estimated at €20 271 per quality-adjusted life year (QUALY), with an 83% probability of being cost-effective.^11^ Similarly, in the Swedish STROKESTOP study, a cost-effectiveness analysis estimated eight fewer strokes and 12 QUALYS per 1000 screenings at an incremental cost of €50 012.^12^ This suggests a good return on investment for initiating AF screening.

In the RoI, the National Cardiovascular Policy 2010 recommended establishment of a screening programme for people aged ≥65 years.^13^ Understanding barriers and enablers to AF screening in primary care is essential to the successful introduction of a national screening programme and to ensure appropriate capacity development.

Primary care has been identified as an effective setting for AF detection and possible screening programmes by the AF-SCREEN International Collaboration.^8^ Primary care has the potential to incorporate AF screening into existing workflows and has the nursing support, depending on the healthcare system in the country in question, to enable screening and manage outcomes, including a direct link to prescribe oral anti-coagulation where required.^8^ In the RoI, GP training incorporates administration and interpretation of 12-lead ECG. Accurate identification of potential facilitators and barriers to AF screening in primary care will inform the development of a national AF screening strategy.

This study aimed to identify the facilitators and barriers to the introduction of an AF screening programme in primary care from the perspectives of GPs, through one-to-one interviews, to inform a pilot AF screening programme.

## Method

This qualitative descriptive study was conducted to inform a pilot AF screening study in the Cork and Kerry region of the RoI.^14^ The study adopted a qualitative descriptive design to specifically identify the facilitators and barriers to AF screening.

### Sampling

GPs were selected from a defined geographic area of Cork city in the RoI. The total population for this area on the north side of Cork city is approximately 42 000 and includes electoral divisions that are among the most deprived nationally on social deprivation indexes.^15^ A list of 54 GPs from 25 practices in the area was obtained from the Health Service Executive (HSE). This list included the names and addresses of practices and GPs, and searches for telephone numbers were made via Google. The practices were contacted via telephone by two members of the study team, AC and FB, on two occasions in November and December 2019, and invited to participate. Reasons for non-participation were not recorded. Of the 54 GPs who were invited to participate, five were ineligible as they were not in practice, leaving 49 eligible GPs.

### Data collection

A structured topic guide (see Supplementary materials) was developed by the study team to explore the barriers and facilitators to AF screening in primary care, and attitudes towards the introduction of an AF screening programme in primary care in the RoI. The topic guide had a total of nine questions. Eight one-to-one interviews were conducted in person with GPs at their practices in January 2020. Interviews were conducted by one of two members of the study team (AC or FB). The interviews were short in duration owing to the highly focused nature of the study and the limited themes. The interviews were audio-recorded and transcribed verbatim.

### Data analysis

Framework analysis was used to analyse the data. This is a method suitable for projects where pre-specified objectives are required. For the purpose of this highly focused study, the themes were pre-determined. The following five-step process was adopted by the framework analysis: familiarisation; identifying a thematic framework; indexing; charting; mapping; and interpretation.^16^ Three members of the research team conducted the initial analysis of the first two interviews line by line and applied codes. The remaining interviews were analysed and coded by two members of the team (AC and FB) under the supervision of a third researcher who is experienced in qualitative research (CPB). The coding structure was compared and any discrepancies in the coding were discussed by these three members of the research team. The codes from each transcript were grouped into sub-themes.

## Results

A total of eight GPs from five practices (five urban and three rural or village) completed the interviews. Four male and four female GPs were interviewed, all of whom were from group practices. The number of years in practice of the GPs ranged from 13–37 years, with an average number of years in practice of 25. The interviews lasted an average of 10 minutes, ranging from 6–14 minutes (Tables 1 2).

**Table 1.** The characteristics of the participants

	Sex	Years in practice	Rural or urban
**Participant 1**	Female	20	Urban
**Participant 2**	Female	15	Urban
**Participant 3**	Female	19	Urban
**Participant 4**	Male	36	Urban
**Participant 5**	Male	13	Rural
**Participant 6**	Male	37	Rural
**Participant 7**	Female	27	Rural
**Participant 8**	Male	36	Urban

**Table 2.** The main themes, sub-themes, and codes

Themes	Sub-themes	Codes
**Facilitators**	Patient facilitators	Education and awareness
	Practice facilitators	Additional staffTraining
	GP facilitators	Prompts and visual aidsProgramme structureReferral pathway
**Barriers**	Patient barriers	CostAccess
	Practice barriers	CostECG machine availabilityDivision of labourDigital record-keeping
	GP barriers	Time limitationsGuideline complexityKnowledge base
**Attitudes**	Willingness to facilitate screening	
	Priority ranking	

ECG = electrocardiogram.

The following three principal pre-determined themes, which informed the topic guide, were discussed: facilitators; barriers; and attitudes to AF screening. The coding of transcripts allowed for more detailed sub-themes to emerge. The sub-themes identified were typically present in the codified transcripts of at least three of the eight GPs interviewed. These included the following: patient facilitators; practice facilitators; GP facilitators; patient barriers; practice barriers; GP barriers; willingness to facilitate; and priority ranking. Patient facilitators and barriers are issues identified by the participants that could directly impact the participation of a patient in an AF screening programme. GP facilitators are issues that could directly impact the participation of a GP. Practice facilitators are issues that could have an impact at a practice level and impact the participation of the wider practice staff or facilities, as identified by the participants. For some themes, for example, time limitations and division of labour, most GPs expressed reservations around AF screening. Other less common concerns included the lack of availability of an ECG in the practice; that is, highlighting material resource constraints as a barrier to participation in an AF screening programme.

The codes that were applied to the data were education, awareness, additional staff, training, time limitations, programme structure, referral pathway, cost, access, guideline complexity, division of labour, digital record-keeping, knowledge base, practice ECG availability, prompts and visual aids.

### Facilitators

#### Patient facilitators

##### Education and awareness

The GPs interviewed identified patient awareness and education as a potential facilitator to AF screening. They discussed methods, such as public awareness campaigns and educational posters, as potential facilitators to engage patients, which they found in their experience to be beneficial. They also discussed existing public awareness campaigns in relation to AF screening. Public awareness was clearly linked by participants to patients presenting for screening. The value placed on public awareness as a facilitator was also evident in the comments of the participants:

Participant 3: *'I think it’s very effective when people hear stuff on the radio or see stuff on the TV, it’s a big difference, they’re more invested then …'*

Participant 7: *'I suppose public awareness, that it’s out there in the public. That the public are aware to try and reach the most vulnerable ….'*

Participant 8: *'I suppose public awareness would be an important factor, if there was a public awareness campaign with posters educating the patients.'*

### Practice facilitators

#### Additional staff or training

Additional or dedicated staff and/or training of staff in the practice to enable the screening process was identified as a facilitator by seven of the participants. The participants had concerns around staffing for a potential AF screening programme. Availability of staff was cited as a potential facilitator to screening as it would mitigate the issue of the GP workload. In particular, the participants discussed having appropriate access to a dedicated nurse resource:

Participant 8: *'Now, if the screening programme for AF was resourced properly i.e., a nurse dedicated.'*

Participant 8: *'Funding for a dedicated nurse, that would be ideal.'*

Participant 3: *'So, practice nurse training, GP training …'*

### GP facilitators

#### Prompts and visual aids

The GPs felt that prompts and visual aids integrated into the computer system could be a significant potential facilitator of screening:

Participant 6: *'Prompts, and it needs to be integrated into the system.'*

Participant 2: *'They’re the main things I suppose that I can think of, the prompts, having the information pre-packed.'*

Participant 6: *'Something up on the screen …'*

#### Programme structure and referral pathway

Programme structure and referral pathway was an ongoing area of discussion throughout the interviews. All of the participants discussed this aspect of screening, which presented itself in different ways through the data. Participants recommended a clear programme structure, enabling screening with integrated agreed referral pathways for screen-detected AF:

Participant 1: '*Well, I suppose it’s like having a definite structure to it. You know, so that is really structured.'*

Participant 3: *'…. just having a clear referral pathway for GPs that’s agreed with secondary care …'*

Participant 4: *' ... there needs to be a structured pathway …'*

Participant 6: *'And access to the AF clinics would be fantastic, that we could have access to them fairly quickly and access to cardiology.'*

### Barriers

#### Patient, practice, and GP barriers

##### Cost

Cost was identified by the participants as a potential barrier to AF screening. In primary care, this would include capacity of the practice and associated costs, and potential patient costs. For example, potential costs incurred by the practice included costs for equipment required for screening such as upgrade of computer systems or clinical equipment (for example, ECG machine). The potential screening costs for patients not entitled to GP care free at the point of delivery was also identified as a potential barrier:

Participant 1: *'We don’t have an ECG machine at present … it is cost that is the issue with it.'*

Participant 6: *'... 'tis all money as well, I have three or four computers that won’t cope with the new systems.'*

Participant 3: *'... cost can be a barrier for them* [the patients] *if they’re between 50 and 70, cost is definitely a barrier for people I think.'*

##### Access

Cost to patients of participating in AF screening was identified as an access barrier. The participants also discussed access to secondary care or specialist services as another potential barrier. This related particularly to potentially vulnerable populations who may not regularly attend primary care and may be missed:

Participant 3: *'… plenty people whose blood pressure and pulse haven’t been checked in a long time … they’re the people you’re probably really interested in getting in …'*

### Practice barriers

#### ECG machine availability

Some of the GPs discussed the lack of availability of an ECG at their practices as a barrier to AF screening owing to its requirement to confirm a clinical diagnosis:

Participant 1: *'The first thing that springs to mind is that we don’t have an ECG machine at present.'*

Participant 2: *'We don’t have a full ECG machine here to date … but we might have to invest.'*

#### Division of labour

A potential solution that participants suggested to mitigate the problem of limited GP time was division of labour, such as the assistance of practice nurses and other healthcare practitioners in the AF screening process:

Participant 2: *'Maybe to involve the practice nurse if possible, in terms of time …'*

Participant 4: *'Obviously, you take nurses’ time ...'*

#### Digital record-keeping

Participants expressed concern about additional work that would be involved in screening programmes on top of their existing paperwork load. Digital record-keeping was identified as a potential facilitator to mitigate this barrier:

Participant 7: *'I’ll be totally honest with you, what puts me off would be the admin and paperwork and having to sit down at a computer and put in figures and that would really I would be really put off by that.'*

### GP barriers

#### Time limitations

Time constraints were discussed throughout all the interviews. There were very conflicting views on time. Many of the GPs interviewed cited time as a potential barrier to AF screening owing to the already short duration of consultations and the difficulty of trying to facilitate other medical issues. Others expressed an alternative view, pointing out the ease of AF screening and the little time taken to screen and how it could easily be accommodated within a consultation. The participants identified that, while AF screening using the 1-lead ECG was relatively quick, actively managing newly diagnosed AF in clinical practice is a much more time-consuming and difficult task:

Participant 1: *'I suppose everything come down to time …'*

Participant 3: *'... the screening is not complicated, it’s relatively straightforward, not time-consuming in my experience.'*

Participant 6: *'Time is a problem. We’re absolutely inundated.'*

#### Guideline complexity

Clear guidelines for a screening programme and management of screen-positive cases are needed. The GPs discussed the complexity of navigating guidelines around detection of new AF cases and the need for simple, agreed guidelines:

Participant 5: *'... if there was guideline, international guidelines, NICE* [The National Institute for Health and Care Excellence] *guidelines, any guideline that would help us to look out for AF …'*

Participant 3: *'... having a clear algorithm for GPs that’s agreed with secondary care.'*

#### Knowledge base

There was recurring commentary on the knowledge base of GPs on AF. The participants discussed reviewing their own learning needs to ensure they were up to date. Training regarding referral pathways was also discussed:

Participant 5*: 'Lack of awareness of the condition …”* Interviewer: '*From the healthcare professionals’ perspective?* Participant 5: *'Yes, absolutely*.'

Participant 2: *'I suppose in terms of knowledge base its I suppose it’s just to provide some up-to-date information on screening, to the GPs ahead of time.'*

### Attitudes

The attitudes of the participants towards AF screening were cautiously positive, with all participants willing to facilitate it within their practices, if barriers could be addressed. A majority of participants ranked AF screening and/or stroke prevention high in terms of their healthcare priorities, mostly because of the devastating effect of stroke owing to unidentified AF and the ease with which AF can be detected.

Participant 3: *'It’s so catastrophic and we’ve had so many patients … it’s an awful when you hear someone’s had a stroke. It’s so devastating so I think it’s a huge priority.'*

Participant 4:*'I think we are ideally placed really…'*

Interviewer: *'Where does stroke prevention or AF detection rank for you in terms of healthcare priority?'*

Participant 6: *'It would be number one …'*

## Discussion

### Summary

With a global ageing population, stroke prevention is a major healthcare priority.^17^ This sentiment was echoed by the participants of this study who placed stroke prevention and AF screening high in terms of healthcare priority. Despite multiple barriers facing GPs, there was significant enthusiasm for AF screening. This study has highlighted some of the main barriers to AF screening in primary care, such as time and resources, and also some of the facilitators to overcome these barriers. The study was undertaken before the COVID-19 global pandemic, and it may be of further interest to conduct a similar study in a post-pandemic climate.

While there is national and international consensus that AF screening is valuable,^8,13^ what is less clear is the optimal mode and location. Primary care has been proposed as a potential location for AF screening,^8^ and the willingness of the participants suggested that there is sufficient buy-in to support this proposal. Many of the participants reported general practice as being ideally placed to conduct AF screening. The current study has highlighted the enthusiasm of participating GPs to engage in an AF screening programme and has explored where some of the gaps exist in terms of feasibility for GPs. It has also highlighted some potential reasons as to why an AF screening programme in primary care may not yet be feasible. Time and resources would need to be addressed in order to make such a programme feasible for GPs. Simple, clear, and definite clinical guidelines have been identified as essential to accompany any future screening programme. This was reflected in many of the GPs' discussions around adequate guidelines and algorithms. This sentiment is echoed in the current ESC guidelines for the management of AF, which recommend that an optimal referral pathway for screening positive cases is essential in the management of patients with confirmed AF.^6^ The results of this study were incorporated into a pilot AF screening programme in the RoI.^14^ In terms of time, the Kardia mobile one-lead ECG device was utilised owing to its ability to provide a reading in 30 seconds. The clinical report form was kept short, at one page, to minimise time spent on administration. Recommendations were made to GPs to combine the AF screening with existing workflows such as COVID-19 vaccination. A referral pathway for newly diagnosed AF was included in the standard operating procedure to include a 12-lead ECG to confirm the diagnosis and onward referral for echocardiogram with an agreed local provider. Patient information on AF was provided to GPs for distribution to newly diagnosed patients, such as *Live Well with AF* booklets, which were provided by Irish Heart Foundation.

### Comparison with existing literature

A questionnaire-based study in the UK conducted in general practice also identified an enthusiasm for AF screening and found similar barriers such as time, workload, capacity, and training.^18^ Another qualitative UK-based study, using focus groups with GPs, pharmacists, and patients, also identified similar barriers, such as patient knowledge and awareness, with patients favouring general practice over community pharmacy for AF detection.^19^ The perspectives of GPs in this study revealed nuances associated with the facilitators and barriers to AF screening, such as time as a barrier and the specific time needs that are generated because of AF screening: while the screening itself is relatively quick to administer, the time taken up managing and referring the patient once diagnosed is where much time is spent. A formal screening programme would also require mechanisms to detect those who have screen-detected AF who do not attend for further testing, or intervention around risk prevention and management.

### Strengths and limitations

The main strength of this study is its qualitative approach to establish a deeper understanding of GP perspectives on facilitators and barriers to AF screening. Understanding these barriers and facilitators to AF screening is essential to design a screening programme that will engage primary care providers. This is the first study, to the authors' knowledge, to qualitatively investigate AF screening from the perspectives of GPs in the RoI.

Framework analysis can be used deductively where pre-determined themes are being explored. This study, with its highly focused, pre-specified objectives and themes, lent itself well to this methodology. However, there are some limitations to the study. First, the participants were all based in one geographic area in Cork, RoI, and there may be different barriers and facilitators to screening in other regions. In an attempt to overcome this, GPs were included in both urban and rural locations. The representativeness of the participating practices in terms of AF prevalence is difficult to estimate and could limit the generalisability of the results. Owing to the lack of universal GP provision in the RoI, it is difficult to estimate the public and private mix of health care, and there is a lack of a clear denominator. The low participation in the study means that all the facilitators and barriers may not have been fully determined. This may be as a result of the manner in which potential participants were contacted; that is, via phone calls to the practice rather than direct contact made with the GPs. The GPs who participated expressed an interest in the topic so are likely enthusiastic about the prospect of AF screening, which may have skewed the results. Another limitation is that only GPs were interviewed, and the views of other healthcare professionals and patients will be important to the success of an AF screening programme. The interviews were short in length at approximately 10 minutes per participant. This was owing to time constraints with participants' workload and the tight focus of the study. The topic guide had a total of nine questions and was designed specifically to investigate facilitators and barriers to AF screening. The interviews took place before the COVID-19 global pandemic; GPs' views in a post-pandemic climate may differ owing to increased time restraints and pressure within the primary care system. Interpretation of 12-lead ECG could be a potential limitation of the study as there will likely be variability in GPs' ability to interpret results; however, unless there is significant tachycardia, AF detection will be relatively straightforward. Potential detection of other cardiovascular diagnoses was not investigated as part of this study, but any issues arising from this can be referred to cardiology where appropriate. This is an area of research that warrants further investigation.

### Implications for research and practice

Globally, as a result of an ageing population, the incidence of stroke owing to AF is rising.^20^ AF meets many of the Wilson and Jungner criteria as a condition suitable for screening.^21^ Early detection can prevent stroke and mitigate some associated morbidity, mortality, and economic costs. One of the current criteria not yet met relates to treatment for AF, which remains suboptimal. This sentiment was reflected in the views of the GPs who expressed the need for guidelines for the treatment of newly identified AF. They also discussed reviewing their learning needs, suggesting that while competent in the treatment of AF, some may not have the confidence to initiate treatment. National AF screening programmes that are conducted at primary care level should include GPs as one of the key stakeholders alongside patients, carers, patient support groups, nurses, secondary care, cardiologists, and public health. This study identified a lack of appropriate referral pathways, which would suggest that all potential stakeholders within an AF screening programme would require alignment ahead of implementation. Current ESC guidelines recommend a structured referral pathway leading to further clinical evaluation, diagnosis, and management of screen-detected cases.^6^ The views of the GPs in this current study suggest that lack of referral pathways is a significant barrier to AF screening in primary care in the RoI. Incorporating and resourcing an adequate referral pathway into an AF screening programme in the RoI would be fundamental to its success. Another implication to consider when implementing an AF screening programme is cost-effectiveness, which has been incorporated into the follow-on pilot AF screening programme.

In conclusion, this study investigated the attitudes of GPs to an AF screening programme and identified possible barriers and facilitators to such a programme. The findings of this qualitative research will help to anticipate barriers, develop screening and clinical care pathways, and inform policymakers on the development of a national AF screening programme. The results were incorporated into and used to inform a pilot AF screening study in the Cork and Kerry region in the RoI.^14^
